# Medical behaviours and medication adherence of older hypertensive patients in different medical insurance programs in Beijing, China: a cross-sectional study

**DOI:** 10.1186/s12877-023-04476-y

**Published:** 2023-12-20

**Authors:** Lan Lan, Pengcheng Hai, Jiawei Luo, Rui Li, Yilong Wang

**Affiliations:** 1 IT Center, Beijing Tiantan Hospital, Capital Medical University, Beijing, 100070 China; 2 Department of Internal Medicine, Beijing Chaoyang Hospital, Capital Medical University, Beijing, 100020 China; 3 West China Biomedical Big Data Center, West China Hospital, Sichuan University, Chengdu, 610044 China; 4 Department of Neurology, Beijing Tiantan Hospital, Capital Medical University, Beijing, 100070 China; 5 Chinese Institute for Brain Research, Beijing, 100070 China; 6 National Center for Neurological Disorders, Beijing, 100070 China; 7 Advanced Innovation Center for Human Brain Protection, Capital Medical University, Beijing, 100070 China; 8 China National Clinical Research Center for Neurological Diseases, Beijing, 100070 China; 9 Beijing Laboratory of Oral Health, Capital Medical University, Beijing, 100070 China; 10 Beijing Municipal Key Laboratory of Clinical Epidemiology, Beijing, 100070 China

**Keywords:** Older, Medical behaviour, Medication adherence, Hypertension, Chinese insurance

## Abstract

**Background:**

Grassroots medical institutions are the primary “battlefield” of hypertension management based on hierarchical diagnosis and treatment policies in China. There is a considerable difference in the insured population and reimbursement ratio between different medical insurance programs. The management of hypertension directly affects the development trend of cardiovascular and cerebrovascular diseases.

**Methods:**

To understand the difference between different medical insurance programs regarding the management of older hypertensive patients, all outpatients aged 60 and above with hypertension in the basic medical insurance database of Beijing, China, from April 1, 2019, to January 31, 2020, were included. Medical behaviours included patients’ choice of hospital level, type of hospital, number of hospitals visited, grassroots medical institutions and cross-district visits. Medication adherence was evaluated by calculating the medication possession ratio of antihypertensive medications. First, we adopted a statistical description for medical behaviours and medication adherence. Then, multivariate logistic regression was used to analyse the influencing factors of medication adherence.

**Results:**

This study included 1.29 million patients with Urban Employee Basic Medical Insurance (UEBMI) and 0.31 million patients with Urban‒Rural Resident Basic Medical Insurance (URRBMI). The proportions of patients with UEBMI who chose tertiary hospitals, comprehensive hospitals, grassroots medical institutions and cross-district visits were 25.84%, 56.09%, 57.34% and 39.32%, respectively, while those of patients with URRBMI were 11.14%, 60.59%, 81.28% and 6.07%, respectively. The medication adherence rates of men and women taking one medication were 61.04% and 55.86%, respectively. UEBMI patients who took their medication accounted for 62.36%, while only 40.27% of URRBMI patients adhered to their medication. The percentages of young-old, old-old and oldest-old patients who took their antihypertensive medications were 58.05%, 59.09% and 56.78%, respectively. The adherence to taking ≥ 2 medications (35.47%) was lower than that to taking one medication (58.33%). The medication adherence rates of patients with UEBMI and URRBMI for taking ≥ 2 medications were 37.21% and 27.45%, respectively.

**Conclusions:**

Patients with UEBMI were more inclined to choose tertiary hospitals and cross-district visits than patients with URRBMI. The adherence of patients with UEBMI was better than that of patients with URRBMI in China.

**Supplementary Information:**

The online version contains supplementary material available at 10.1186/s12877-023-04476-y.

## Introduction

The pace of population ageing is much faster than in the past, and the challenge of ensuring the health and social systems of older individuals during this demographic transition is experienced worldwide, as reported by the World Health Organization (WHO) [1]. Due to the absolute size of China’s older population (by 2020, the number of people aged 65 and above reached 190 million, accounting for 13.5% [2]), rapid development, lower life expectancy per capita than that of developed countries, an enormous gap between the supply and demand of older care, and unbalanced urban‒rural and regional development, China’s population ageing status is severe and faces more complex problems.

Hypertension in older people is a major factor that endangers survival and quality of life. Active treatment can substantially reduce the risk of moderate and major cardiovascular stroke events. In 2019, more than 1 billion people suffered from hypertension (82% of all hypertensive patients worldwide) in low-income and middle-income areas [3]. According to the Chinese Cardiovascular Health and Disease Report 2019 released by the Chinese Cardiovascular Center, the number of Chinese adult hypertensive patients reached 245 million. Among them, the prevalence of hypertension in Beijing was 35.9%, ranking first in the country [4]. Hypertension is relatively common in China, and its prevalence is increasing yearly, but it has not been fully controlled [5].

In 2015, China issued the Guiding Opinions of the General Office of the State Council on Promoting the Construction of a Hierarchical Diagnosis and Treatment System [6], which required that the pilot work of hierarchical diagnosis and treatment of chronic diseases such as hypertension be performed well. Hierarchical diagnosis and treatment refers to grading according to the priority of the disease and the difficulty of treatment. The framework of the hierarchical diagnosis and treatment system includes a community first diagnosis model, two-way referrals, urgent and timely treatment, and linkage between upper and lower levels. The first consultation at the grassroots level is to adhere to the principle of voluntary participation by the masses and encourage patients with common and frequently occurring diseases to seek medical treatment at grassroots medical institutions through policy guidance. Grassroots medical institutions are the primary “ battlefield” of hypertension management, and the management of hypertension directly affects the development trend of cardiovascular and cerebrovascular diseases in China. Compared with other cities in China, Beijing is a large city with high-quality medical resources, and the behaviours of older hypertensive patients in Beijing are worth studying.

It has been estimated that 33 ~ 69% of medication-related hospitalizations in the United States are due to poor medication adherence, with an annual cost of $100 billion [7]. For hypertension, the higher the adherence to treatment is, the lower the risk of hospitalization [8]. Treatment adherence is currently recognized as the key to effective antihypertensive treatment. However, adherence is often ignored in the management of hypertension. The reason is that there is no uniform standard and extremely complex methods of adherence assessment in clinical practice, as well as limitations in clinical practice [9]. Poor medication adherence undermines the expectation of medication treatment and increases the risk of occurrence and development of cardiovascular and cerebrovascular diseases and kidney diseases. Existing evidence shows that up to 90% of hypertensive patients may not insist on treatment, and approximately 50% may stop treatment within one year after diagnosis [10].

There are few studies on the medical behaviours of middle-aged and older hypertensive patients in China. Their data are from sample surveys, which do not reflect the overall situation of a city or region, and the sample size is within 5000 cases [11–13]. There are studies on medication adherence among hypertensive patients from Beijing [14], Shanghai [15], Shanxi [16], Hunan [17], Shaanxi [18] and Hong Kong [19] in China in which patients self-reported their adherence based on a scale. However, there is little research on the results of medication adherence among older hypertensive patients in China. Only the China Health and Retirement Longitudinal Study (CHARLS) in 2015 [20] evaluated the adherence of middle-aged and older hypertensive patients, and a survey based on a scale from Jiangsu [21] evaluated the adherence of older patients to antihypertensive medications in China.

There is a considerable difference in the insured population and reimbursement ratio between different medical insurance programs. Therefore, to understand the difference between different medical insurance programs for the management of older hypertensive patients in China, this study used a large number of claims data to study the medical behaviours and medication adherence of hypertensive patients aged 60 and above to improve the survival and quality of life of older hypertensive patients.

## Methods

### Data and subjects

This study was based on the “Study on the technical standard of capitation for chronic diseases” of the Beijing Medical Insurance Bureau. All outpatients aged 60 and above with hypertension in the basic medical insurance database of Beijing from April 1, 2019, to January 31, 2020, were included. The data were deidentified before analysis.

### Definition

Patient characteristics. According to the WHO age classification standard for older people [22, 23], young-old people are 60 ~ 74 years old, old-old people are 75 ~ 89 years old, and oldest-old people are 90 years old and above. Sex was a binary variable. Medical insurance included 2 major health insurance schemes in China: Urban Employee Basic Medical Insurance (UEBMI) and Urban‒Rural Resident Basic Medical Insurance (URRBMI). The people who participate in UEBMI are mainly employees, including employees of companies, government agencies, public institutions, social organizations, and private noncompany workplaces. The reimbursement ratio for outpatient (emergency) consultations of in-service employees in Beijing has reached 70%, and that for retirees has reached 85%. The reimbursement ratio of community hospitals is 90%. Regarding outpatient reimbursements of over 20,000 Chinese yuan, if medical expenses are incurred again, 60% is reimbursed for in-service employees and 80% for retirees, with no upper limit. The people who participate in URRBMI are mainly urban and rural residents without workplaces. During a medical insurance year, 55% of outpatient (emergency) consultations for urban and rural residents covered by URRBMI are reimbursed at level 1 or below hospitals, and 50% are reimbursed at level 2 and level 3 hospitals; the upper limit is 4500 Chinese yuan.

Medical behaviours. Grassroots medical institutions in China include community health service centres (stations), outpatient departments, clinics, health centres (rooms), infirmaries, nursing stations, student health care centres, and village health rooms. In this study, we set grassroots medical institutions as a binary variable (whether a doctor was seen in a grassroots medical institution). Hospital levels included level 3, level 2, level 1 and no level, whereby the higher the level was, the larger the scale of the hospital. Type of hospital was divided into comprehensive hospitals, traditional Chinese medicine hospitals, specialized hospitals and community hospitals. There are 16 districts in Beijing. If the patient did not visit the hospital in their residential district, it was defined as a cross-district visit (binary: yes or no). The number of hospitals visited during the observation period was set as a categorical variable: 1, 2 and ≥ 3. Multiple visits from the same hospital counted as one.

Medication adherence. Since there are no gold standard methods for adherence evaluation, this study used claims data with low measurement costs to calculate adherence to antihypertensive medications. We used the medication possession ratio (MPR) to analyse adherence to antihypertensive medications, which was defined as the sum of all supply days of antihypertensive medications divided by the days during the observation period (from the first prescription to the last prescription) [24]. The patient had at least 3 outpatient visits and antihypertensive medication prescription records. When we calculated the total supply days of medications, the last outpatient visit was excluded. Antihypertensive medications were divided into five categories: thiazide diuretics, calcium channel blockers, angiotensin-converting enzyme (ACE) inhibitors, angiotensin receptor blockers (ARBs) and beta blockers. We estimated the medication adherence of each type of antihypertensive medication. When calculating the MPR of patients taking ≥ 2 medications, the numerator was the cumulative maximum supply days of medications per visit excluding the last visit, and the denominator remained unchanged. If the MPR was greater than or equal to 0.8, the patient was considered adherent; otherwise, the patient was considered nonadherent [25, 26].

### Statistical analysis

Since it is easy to obtain significant differences using a large sample, we applied a statistical description to show the difference. Multivariate logistic regression was used to analyse the influencing factors, including sex, age, medical insurance program and medical behaviours, for adherence (binary: adherent and nonadherent), where the first visit in our observation time was selected for medical behaviours except for the number of hospitals visited. Odds ratios (ORs) and their 95% confidence intervals (CIs) were obtained from logistic regression. A 95% CI that did not cross 1 indicated statistical significance (*P* < 0.05). Our methods were carried out in accordance with SAMPL guidelines. All analyses were performed with R (Version 4.2.1).

## Results

### Basic characteristics

This study included 1.6 million older hypertensive outpatients in Beijing, China, comprising 1.29 million patients with UEBMI and 0.31 million patients with URRBMI. Less than 50% of the hypertensive patients were older males. Most patients were young-old, while oldest-old patients accounted for 1.89%. Each patient had an average of 13.55 outpatient visits and 288.39 days of observation time in this study. The average medical expense of each outpatient visit was 511.05 Chinese yuan. The visits with antihypertensive medications accounted for 63.89% of outpatient visits, in which the number of medication prescriptions from highest to lowest was calcium channel blockers, ARBs, beta blockers, thiazide diuretics and ACE inhibitors. Table 1 shows information on patients with UEBMI and URRBMI.

**Table 1:** Basic characteristics of older hypertensive patients in Beijing, China

Characteristics	UEBMI	URRBMI	Total
Total patients (%)	1,294,363 (80.63)	311,044 (19.37)	1,605,407 (100.00)
Male patients (%)	645,039 (49.83)	114,901 (36.94)	759,940 (47.34)
Age, patients (%)			
60 ~ 74	886,525 (68.49)	222,599 (71.57)	1,109,124 (69.09)
75 ~ 89	383,203 (29.61)	82,658 (26.57)	465,861 (29.02)
≥ 90	24,635 (1.90)	5787 (1.86)	30,422 (1.89)
Number of outpatient visits			
Total	18,762,328	2,989,784	21,752,112
Each patient	14.50	9.61	13.55
Observation time, days			
Total	378,847,830	84,138,589	462,986,419
Each patient	292.69	270.50	288.39
Outpatient expenditures, Chinese yuan			
Total	10,347,701,479	768,666,046	11,116,367,525
Each visit	551.51	257.10	511.05
Antihypertensive medications, visits (%)	11,799,646 (62.89)	2,098,148 (70.18)	13,897,794 (63.89)
Thiazide diuretics	2,060,005 (17.46)	546,382 (26.04)	2,606,387 (18.75)
Calcium channel blockers	7,346,310 (62.26)	1,238,377 (59.02)	8,584,687 (61.77)
ARBs	5,141,735 (43.58)	870,842 (41.51)	6,012,577 (43.26)
ACE inhibitors	930,643 (7.89)	146,684 (6.99)	1,077,327 (7.75)
Beta blockers	3,090,159 (26.19)	372,988 (17.78)	3,463,147 (24.92)

UEBMI, Urban Employee Basic Medical Insurance; URRBMI, Urban‒Rural Resident Basic Medical Insurance; ARBs, angiotensin receptor blockers; ACE, angiotensin-converting enzyme

### Medical behaviours

Compared with URRBMI patients, UEBMI patients tended to choose tertiary hospitals. Regarding the type of hospital, URRBMI patients were slightly more likely to choose a comprehensive hospital. URRBMI patients were more likely to choose grassroots medical institutions, while UEBMI patients were more likely to choose cross-district visits (Table 2). See Table S1, Table S2 and Table S3 for medical behaviours of young-old, old-old, and oldest-old patients.

**Table 2:** Medical behaviours of older hypertensive patients in Beijing, China

Medical behaviours	UEBMI		URRBMI		Total	
	Visit	%	Visit	%	Visit	%
Hospital level						
Level 3	4,849,092	25.84	333,029	11.14	5,182,121	23.82
Level 2	1,824,570	9.72	184,368	6.17	2,008,938	9.24
Level 1	5,619,690	29.95	1,409,167	47.13	7,028,857	32.31
No level	6,468,976	34.48	1,063,220	35.56	7,532,196	34.63
Hospital type						
Comprehensive hospital	10,524,085	56.09	1,811,521	60.59	12,335,606	56.71
Specialized hospital	1,028,185	5.48	54,050	1.81	1,082,235	4.98
Traditional Chinese medicine hospital	2,026,774	10.80	126,433	4.23	2,153,207	9.90
Community hospital	5,183,284	27.63	997,780	33.37	6,181,064	28.42
Grassroots medical institution visit, yes	10,758,703	57.34	2,430,141	81.28	13,188,844	60.63
Cross-district visit, yes	7,377,473	39.32	181,474	6.07	7,558,947	34.75

UEBMI, Urban Employee Basic Medical Insurance; URRBMI, Urban‒Rural Resident Basic Medical Insurance

### Medication adherence

Regarding adherence to antihypertensive medications, more than half of the older hypertensive patients took their medications. The proportion of UEBMI patients with medication adherence was far higher than that of URRBMI patients, the proportion of males was higher than that of females, and the proportion of oldest-old patients was slightly lower than that of the other two age groups (Table 3). For those taking ≥ 2 antihypertensive medications, their medication adherence was much lower than that of those taking one medication, while differences in medication adherence between different groups were similar to those of the patients taking one medication (Table 4).

**Table 3:** Medication adherence of older hypertensive patients taking one medication in Beijing, China

Group	Medications (Patients)	MPR				Adherence	
		Mean	SD	Median	IQR	Patient	%
Total	1,314,874	0.788	0.243	0.895	0.403	767,015	58.33
Sex							
Male	627,728	0.802	0.238	0.922	0.378	383,191	61.04
Female	687,146	0.775	0.247	0.866	0.424	383,824	55.86
Age, years							
60 ~ 74	903,387	0.787	0.243	0.890	0.405	524,442	58.05
75 ~ 89	386,739	0.792	0.243	0.905	0.397	228,522	59.09
≥ 90	24,748	0.781	0.244	0.875	0.409	14,051	56.78
Medical insurance							
UEBMI	1,075,493	0.811	0.263	0.688	0.513	670,624	62.36
URRBMI	239,381	0.684	0.232	0.931	0.358	96,391	40.27

MPR, medication possession ratio; SD, standard deviation; IQR, interquartile range; UEBMI, Urban Employee Basic Medical Insurance; URRBMI, Urban‒Rural Resident Basic Medical Insurance

**Table 4:** Medication adherence of older hypertensive patients taking ≥ 2 medications in Beijing, China

Group	Medications (Patients)	MPR				Adherence	
		Mean	SD	Median	IQR	Patient	%
Total	298,151	0.674	0.227	0.681	0.381	105,744	35.47
Sex							
Male	146,772	0.681	0.226	0.691	0.384	53,974	36.77
Female	151,379	0.667	0.227	0.672	0.377	51,770	34.20
Age, years							
60 ~ 74	209,059	0.674	0.226	0.681	0.380	73,952	35.37
75 ~ 89	84,753	0.675	0.228	0.683	0.382	30,280	35.73
≥ 90	4339	0.668	0.228	0.672	0.379	1512	34.85
Medical insurance							
UEBMI	244,775	0.686	0.223	0.698	0.377	91,092	37.21
URRBMI	53,376	0.620	0.235	0.601	0.379	14,652	27.45

MPR, medication possession ratio; SD, standard deviation; IQR, interquartile range; UEBMI, Urban Employee Basic Medical Insurance; URRBMI, Urban‒Rural Resident Basic Medical Insurance

The MPRs of the five types of antihypertensive medications were different, whereby calcium channel blockers were the most common and beta blockers were the least common. Men generally had a higher MPR than women, UEBMI patients had a higher MPR than URRBMI patients, and oldest-old patients had a lower MPR than the other two age groups (Table 5).

**Table 5:** MPRs of five antihypertensive medications used by older hypertensive patients

Group	Thiazide diuretics		Calcium channel blockers		ARBs		ACE inhibitors		Beta blockers	
	Medications (Patients)	MPR	Medications (Patients)	MPR	Medications (Patients)	MPR	Medications (Patients)	MPR	Medications (Patients)	MPR
Total	318,522	0.679	987,266	0.684	709,710	0.676	129,266	0.660	438,888	0.617
Sex										
Male	157,015	0.686	470,040	0.696	344,675	0.685	71,500	0.667	220,299	0.629
Female	161,507	0.672	517,226	0.672	365,035	0.667	57,766	0.652	218,589	0.604
Age										
60 ~ 74	214,976	0.679	672,513	0.685	501,212	0.674	92,486	0.664	295,822	0.622
75 ~ 89	97,725	0.680	295,956	0.682	197,767	0.681	34,605	0.650	135,404	0.607
≥ 90	5821	0.671	18,797	0.676	10,731	0.672	2175	0.652	7662	0.598
Medical insurance										
UEBMI	247,174	0.691	819,687	0.704	593,120	0.692	110,058	0.681	386,203	0.632
URRBMI	71,348	0.639	167,579	0.583	116,590	0.596	19,208	0.543	52,685	0.508

MPR, medication possession ratio; ARBs, angiotensin receptor blockers; ACE, angiotensin-converting enzyme; UEBMI, Urban Employee Basic Medical Insurance; URRBMI, Urban‒Rural Resident Basic Medical Insurance

Table 6 shows that there were differences in medication adherence to the five types of antihypertensive medications. Calcium channel blockers had the best adherence, which was 8.87% higher than the lowest adherence of beta blockers. The proportion of men with medication adherence was 3.29% higher than that of women on average. The proportion of UEBMI patients who were adherent to their antihypertensive medications was 15.46% higher than the proportion of URRBMI patients. There was no significant difference in the proportion of medication adherence among different age groups. In general, the proportion of patients aged 90 and above who adhered to their medication was smaller than that of the other two age groups.

**Table 6:** Medication adherence to five antihypertensive medications among older hypertensive patients

Group	Thiazide diuretics		Calcium channel blockers		ARBs		ACE inhibitors		Beta blockers	
	Adherence (Patients)	Adherence (%)	Adherence (Patients)	Adherence (%)	Adherence (Patients)	Adherence (%)	Adherence (Patients)	Adherence (%)	Adherence (Patients)	Adherence (%)
Total	118,172	37.10	369,995	37.48	254,362	35.84	44,995	34.81	125,551	28.61
Sex										
Male	60,310	38.41	187,067	39.80	129,507	37.57	25,576	35.77	67,292	30.55
Female	57,862	35.83	182,928	35.37	124,855	34.20	19,419	33.62	58,259	26.65
Age										
60 ~ 74	79,378	36.92	253,451	37.69	177,865	35.49	32,787	35.45	86,559	29.26
75 ~ 89	36,669	37.52	109,810	37.10	72,719	36.77	11,500	33.23	36,980	27.31
≥ 90	2125	36.51	6734	35.82	3778	35.21	708	32.55	2012	26.26
Medical insurance										
UEBMI	96,075	38.87	333,457	40.68	226,923	38.26	41,654	37.85	117,583	30.45
URRBMI	22,097	30.97	36,538	21.80	27,439	23.53	3341	17.39	7968	15.12

ARBs, angiotensin receptor blockers; ACE, angiotensin-converting enzyme; UEBMI, Urban Employee Basic Medical Insurance; URRBMI, Urban‒Rural Resident Basic Medical Insurance

For the five antihypertensive medications, the adherence of the old-old group was better or worse than that of the young-old group, but the adherence of the oldest-old group was worse than that of the young-old group. The type of medical insurance had the greatest impact on adherence. Patients who chose a tertiary hospital had higher adherence than those who did not. Patients who chose specialized hospitals and comprehensive hospitals had higher adherence than those who chose community hospitals, while patients who chose traditional Chinese medicine hospitals had lower adherence than those who chose community hospitals. Patients who chose grassroots medical institutions had higher adherence. The level of adherence of patients who chose a hospital outside of their district was inconsistent among the five types of antihypertensive medications (Table 7).

**Table 7:** Factors that influenced adherence to five antihypertensive medications according to multivariate logistic regression

Variable	Thiazide diuretics	Calcium channel blockers	ARBs	ACE inhibitors	Beta blockers
Male, ref = Female	1.09^*^ (1.07,1.11)	1.15^*^ (1.14,1.16)	1.11^*^ (1.1,1.12)	1.02 (1.00,1.05)	1.14^*^ (1.12,1.15)
Age, ref = 60 ~ 74					
75 ~ 89	1.01 (0.99,1.02)	0.95^*^ (0.94,0.96)	1.03^*^ (1.02,1.04)	0.89^*^ (0.87,0.91)	0.89^*^ (0.88,0.90)
≥ 90	0.97 (0.92,1.02)	0.90^*^ (0.88,0.93)	0.98 (0.94,1.02)	0.86^*^ (0.79,0.94)	0.87^*^ (0.82,0.91)
UEBMI, ref = URRBMI	1.38^*^ (1.36,1.41)	2.39^*^ (2.36,2.42)	1.92^*^ (1.89,1.95)	2.67^*^ (2.57,2.78)	2.22^*^ (2.16,2.28)
Hospital level, ref = Level 1					
Level 3	1.24^*^ (1.19,1.29)	1.25^*^ (1.23,1.27)	1.27^*^ (1.24,1.30)	1.55^*^ (1.47,1.65)	1.70^*^ (1.64,1.75)
Level 2	0.96 (0.92,1.00)	1.02 (0.99,1.04)	0.99 (0.96,1.02)	1.09^*^ (1.02,1.16)	1.06^*^ (1.02,1.10)
No level	1.20^*^ (1.16,1.25)	1.17^*^ (1.15,1.19)	1.17^*^ (1.15,1.20)	1.20^*^ (1.15,1.26)	1.10^*^ (1.07,1.13)
Hospital type, ref = Community hospital					
Comprehensive hospital	1.10^*^ (1.06,1.13)	1.11^*^ (1.09,1.13)	1.09^*^ (1.07,1.12)	1.03 (0.99,1.08)	1.05^*^ (1.02,1.08)
Specialized hospital	1.30^*^ (1.24,1.37)	1.26^*^ (1.23,1.29)	1.24^*^ (1.20,1.29)	1.25^*^ (1.17,1.34)	1.24^*^ (1.19,1.29)
Traditional Chinese medicine hospital	0.94^*^ (0.90,0.98)	0.99 (0.97,1.02)	0.92^*^ (0.89,0.95)	0.77^*^ (0.72,0.82)	0.82^*^ (0.79,0.86)
Grassroots medical institution visit, yes	1.08^*^ (1.04,1.12)	1.07^*^ (1.05,1.09)	1.05^*^ (1.02,1.08)	1.10^*^ (1.03,1.16)	1.05^*^ (1.01,1.08)
Cross-district visit, yes	0.99 (0.97,1.01)	0.98^*^ (0.97,0.99)	1.01 (1.00,1.02)	1.13^*^ (1.11,1.16)	1.05^*^ (1.04,1.07)

ARBs, angiotensin receptor blockers; ACE, angiotensin-converting enzyme; UEBMI, Urban Employee Basic Medical Insurance; URRBMI, Urban‒Rural Resident Basic Medical Insurance. Outside the bracket is the odds ratio (OR), and inside the bracket is its 95% confidence interval (CI). * statistically significant

In general, sex, age, medical insurance program, and medical behaviours affected adherence. The more hospitals visited, the better the patients’ adherence. Regarding taking ≥ 2 antihypertensive medications, all factors except for age had statistical significance. The relationship between the number of hospitals visited and adherence was different among people taking one medication. In other words, these factors had different effects on people who took one medication and those who took ≥ 2 medications (Table 8).

**Table 8:** Factors that influenced medication adherence using multivariate logistic regression

Variable	One medication	≥ 2 medications
Male, ref = Female	1.18^*^(1.18,1.19)	1.09^*^(1.07,1.11)
Age, ref = 60 ~ 74		
75 ~ 89	1.01^*^(1.00,1.02)	1.00(0.98,1.02)
≥ 90	0.94^*^(0.92,0.97)	0.97(0.91,1.03)
UEBMI, ref = URRBMI	2.25^*^(2.22,2.27)	1.50^*^(1.47,1.54)
Hospital level, ref = Level 1		
Level 3	1.39^*^(1.36,1.41)	1.20^*^(1.16,1.25)
Level 2	1.18^*^(1.15,1.20)	0.95^*^(0.91,0.99)
No level	1.13^*^(1.11,1.15)	1.21^*^(1.16,1.25)
Hospital type, ref = Community hospital		
Comprehensive hospital	1.10^*^(1.08,1.12)	1.11^*^(1.07,1.15)
Specialized hospital	1.14^*^(1.11,1.17)	1.29^*^(1.22,1.35)
Traditional Chinese medicine hospital	0.89^*^(0.87,0.91)	0.95^*^(0.91,1.00)
Grassroots medical institution visit, yes	1.19^*^(1.17,1.21)	1.05^*^(1.01,1.09)
Cross-district visit, yes	0.97^*^(0.96,0.98)	1.02^*^(1.00,1.04)
Number of hospitals visited, ref = 1		
2	1.27^*^(1.26,1.28)	0.98^*^(0.96,0.99)
≥ 3	2.00^*^(1.97,2.02)	1.10^*^(1.06,1.14)

UEBMI, Urban Employee Basic Medical Insurance; URRBMI, Urban‒Rural Resident Basic Medical Insurance. Outside the bracket is the odds ratio (OR), and inside the bracket is its 95% confidence interval (CI). * statistically significant

## Discussion

Based on claims data, this study analysed the medical behaviours and medication adherence of older hypertensive patients in Beijing, China, and revealed the current state of older hypertensive patients with different basic medical insurance schemes, providing a scientific basis for better management of hypertensive patients.

According to the Guiding Opinions of the General Office of the State Council on Promoting the Construction of a Hierarchical Diagnosis and Treatment System [6], grassroots medical institutions should provide medical services for patients with chronic diseases with a clear diagnosis and stable condition. The relevant national departments have successively issued preferential policies for the first diagnosis and reimbursement of chronic diseases at the grassroots level, aiming to further guide patients with chronic diseases to see doctors at the grassroots level. CHARLS showed that 57.63% of older people choose grassroots medical institutions when they visit a doctor in China [27]. In this study, 60.63% of older people with hypertension chose grassroots medical institutions for outpatient treatment. More than half of the older patients with hypertension chose comprehensive hospitals, and the proportion that chose tertiary hospitals was not low, which undoubtedly increases the burden on large hospitals crowded with visitors. It is necessary to further guide patients with chronic diseases such as hypertension to seek treatment in grassroots medical institutions. The proportion of UEBMI patients who chose tertiary hospitals and cross-district visits was higher than that of URRBMI patients, which may be related to the higher proportion of UEBMI reimbursement than that of URRBMI, indicating that the medical insurance policy has a great impact on patients’ medical behaviours. The difference between UEBMI and URRBMI patients aged 90 and above in choosing tertiary hospitals, grassroots medical institutions and cross-district visits was smaller than that of the other two age groups.

The medication adherence rates of hypertensive patients in China differ greatly. A survey from Beijing showed that 61.3% of 318 rural hypertensive patients had medication adherence, and 38.7% had no medication adherence [14]. A survey of outpatients in a hospital in Shanghai showed that 51.7% of the patients had high adherence to antihypertensive medications, 22.0% had moderate adherence, and 26.3% had low adherence [15]. A Hong Kong survey showed that 65.1% of patients had good adherence to antihypertensive medications, while 32.6% had poor adherence [19]. However, a survey in Jinzhong, Shanxi, showed that 21.3% of rural hypertensive patients insisted on taking medications, while 78.7% did not [16]. A survey in Changsha, Hunan, showed that 63.6% of hypertensive patients had low adherence, 29.5% had moderate adherence and 7.6% had high adherence [17]. According to a survey conducted at a hospital in Xi’an, Shaanxi, 27.46% of the patients complied with antihypertensive treatment [18]. The above medication adherence results were lower than the antihypertensive medication adherence results (75%) of Chinese immigrants to the United States [28].

With respect to the medication adherence of older patients with hypertension, a survey in Suzhou, Jiangsu, showed that 34.2% of older patients had good adherence to antihypertensive medications, while 65.8% had poor adherence [21]. The CHARLS showed that 77.2% of middle-aged and older hypertensive patients reported adherence to medication [20]. A systematic review showed that the self-reported medication adherence rate of hypertension patients over 60 years of age was 68.86%, and the adherence rate of patients in Western countries (e.g., Europe and the United States) was substantially higher than that of other patients [29]. Li et al. reported that the medication adherence of male and female older hypertensive patients who immigrated from China to the United States was 69% and 75%, respectively [30]. According to a survey by Hsu et al., 52% of older Chinese Americans insisted on taking antihypertensive medications [31]. These results on medication adherence vary greatly because of different measurement methods and samples. Their medication adherence results were based on self-reports, which is quite different from the calculation methods of this study. It is difficult to directly compare our rate of 58.33% among older hypertensive patients in Beijing with rates in other studies. Based on medical claims data, the results of sampling calculations from Manitoba, Canada, showed that 76.7% of hypertensive patients aged 65 or older were considered adherent when using the prescription-based MPR [32], which was higher than the adherence rate in this study. Our results showed that adherence to multiple medications was worse than adherence to only one medication. The reason may be that when the patient’s condition is severe, multiple antihypertensive medications are needed to control their blood pressure. Once their blood pressure is controlled, the patient may not want to take many medications, leading to poor adherence. This reveals that there is still much room for improvement in medication adherence in China. However, this study is the first to report the results of medication adherence among older patients with hypertension in a whole population of a city based on claims data in China, rather than a sampling survey. Our results are important for better management of older patients with hypertension.

Moreover, there were differences in medication adherence to different antihypertensive medications in this study. Calcium channel blockers have strong antihypertensive effects, good tolerance, no absolute contraindications, and a relatively wide range of applications. Calcium channel blockers are more suitable for simple systolic hypertension, which made adherence to them relatively high. The adherence to beta blockers was relatively low, which may be because the medication is mainly used in patients with a faster heart rate to lower it. The aggregated MPR and adherence rate were lower for multiple medications than for a single medication.

The difference between the two types of medical insurance populations led to differences in medication adherence. There are differences between men and women in the prevalence of hypertension, the effect of antihypertensive medications and the risk of side effects of antihypertensive medications [33], which may lead to differences in medication adherence between the sexes. The adherence of hypertension patients aged 90 and above is worse, which may be due to their own physical limitations. If there is someone to take care of these older patients, their medication adherence may improve.

Tertiary hospitals provide high-level specialized medical and health services in the region. Hospitals without a level have not been evaluated and graded, which can be a complex process, and these hospitals include both large and small hospitals. Compared with level 1 hospitals, patients may trust level 2 hospitals and no-level hospitals more, so their adherence was higher. Compared with community hospitals, patients in comprehensive hospitals and specialized hospitals may have higher adherence because they trust comprehensive hospitals and specialized hospitals more. The reason why the adherence of patients in traditional Chinese medicine hospitals was lower than that in community hospitals may be related to the nature of traditional Chinese medicine hospitals, as they can also prescribe these five antihypertensive medications. The reason for the higher adherence of patients who chose grassroots medical institutions may be that the patients’ condition was relatively stable, and the patients were clear about their needs, so they went directly to a grassroots medical institution that had few people and found it convenient to have medicine prescribed. The factors that affected the adherence of patients taking ≥ 2 medications were more complex. Compared to taking one medication, the factors that influenced the adherence of patients taking ≥ 2 medications had inconsistent effects. For example, the impact of age and medical insurance is decreasing. The role of number of hospitals visited is trending in the opposite direction. The reasons behind this are worth further investigation and research in the future.

Because we did not know the actual days or doses of patients taking medications, this study used the MPR to estimate medication adherence to antihypertensive medications, which may have overestimated the adherence of patients compared with the self-reported medication adherence evaluated by instruments such as the Morisky Medication Adherence Scale (MMAS). Some variables were not included in the establishment of the medical insurance database, such as income, which is an important factor that can affect patient medical behaviours and medication adherence, which is a limitation of this study. This was also a cross-sectional study that did not consider the patient’s first diagnosis of hypertension or subsequent disease development, thus ignoring the impact of the development of hypertension on medical adherence.

In summary, based on claims data, this study investigated the medical behaviours and medication adherence of older hypertensive patients in Beijing, China. Patients with UEBMI had a higher frequency of visits and higher average medical expenses per visit and were more inclined to choose tertiary hospitals and hospitals outside their district than patients with URRBMI. The adherence of patients with UEBMI was better than that of patients with URRBMI.

### Electronic supplementary material

Below is the link to the electronic supplementary material.

**Supplementary: Table S1**. Medical behaviors of hypertensive patients aged 60~74 in Beijing, China. **Table S2**. Medical behaviors of hypertensive patients aged 75~89 in Beijing, China. **Table S3**. Medical behaviors of hypertensive patients aged 90 or older in Beijing, China.

## Data Availability

The data used in the study is not publicly available, but the data used and/or analysed during the current study are available from the corresponding author on reasonable request.
