# Supplementary material for: Medical behaviours and medication adherence of older hypertensive patients in different medical insurance programs in Beijing, China: a cross-sectional study
Source: BMC Geriatr. 2023 Dec 20;23:878. doi: 10.1186/s12877-023-04476-y (PMC10734068; doi:10.1186/s12877-023-04476-y)
Supplement: Supplementary file 1 — Supplementary: Table S1. Medical behaviors of hypertensive patients aged 60~74 in Beijing, China. Table S2. Medical behaviors of hypertensive patients aged 75~89 in Beijing, China. Table S3. Medical behaviors of hypertensive patients aged 90 or older in Beijing, China. [file 12877_2023_4476_MOESM1_ESM.docx]

**Supplementary**

Table S1. Medical behaviors of hypertensive patients aged 60~74 in Beijing, China.

| Medical behaviors | UEBMI | | URRBMI | | Total | |
| --- | --- | --- | --- | --- | --- | --- |
|  | Visit | % | Visit | % | Visit | % |
| Hospital level |  |  |  |  |  |  |
| Level 3 | 3118869 | 24.47 | 222352 | 10.30 | 3341221 | 22.42 |
| Level 2 | 1214364 | 9.53 | 127168 | 5.89 | 1341532 | 9.00 |
| Level 1 | 4002517 | 31.40 | 1058891 | 49.07 | 5061408 | 33.96 |
| No level | 4410046 | 34.60 | 749339 | 34.73 | 5159385 | 34.62 |
| Hospital type |  |  |  |  |  |  |
| Comprehensive hospital | 7148703 | 56.09 | 1326575 | 61.48 | 8475278 | 56.87 |
| Specialized hospital | 654081 | 5.13 | 35845 | 1.66 | 689926 | 4.63 |
| Traditional Chinese medicine hospital | 1385120 | 10.87 | 86017 | 3.99 | 1471137 | 9.87 |
| Community hospital | 3557892 | 27.91 | 709313 | 32.87 | 4267205 | 28.63 |
| Grassroots medical institution, yes | 7571076 | 59.40 | 1781050 | 82.54 | 9352126 | 62.75 |
| Cross-district, yes | 5092632 | 39.96 | 111731 | 5.18 | 5204363 | 34.92 |

UEBMI, Urban Employee Basic Medical Insurance; URRBMI, Urban-Rural Resident Basic Medical Insurance.

Table S2. Medical behaviors of hypertensive patients aged 75~89 in Beijing, China.

| Medical behaviors | UEBMI | | URRBMI | | Total | |
| --- | --- | --- | --- | --- | --- | --- |
|  | Visit | % | Visit | % | Visit | % |
| Hospital level |  |  |  |  |  |  |
| Level 3 | 1635916 | 28.79 | 102604 | 13.11 | 1738520 | 26.89 |
| Level 2 | 574672 | 10.11 | 53089 | 6.78 | 627761 | 9.71 |
| Level 1 | 1531366 | 26.95 | 331517 | 42.36 | 1862883 | 28.82 |
| No level | 1940203 | 34.15 | 295457 | 37.75 | 2235660 | 34.58 |
| Hospital type |  |  |  |  |  |  |
| Comprehensive hospital | 3191943 | 56.17 | 456058 | 58.27 | 3648001 | 56.43 |
| Specialized hospital | 349079 | 6.14 | 16382 | 2.09 | 365461 | 5.65 |
| Traditional Chinese medicine hospital | 605122 | 10.65 | 37669 | 4.81 | 642791 | 9.94 |
| Community hospital | 1536013 | 27.03 | 272558 | 34.82 | 1808571 | 27.98 |
| Grassroots medical institution, yes | 3014714 | 53.06 | 613263 | 78.36 | 3627977 | 56.12 |
| Cross-district, yes | 2152202 | 37.88 | 61472 | 7.85 | 2213674 | 34.24 |

UEBMI, Urban Employee Basic Medical Insurance; URRBMI, Urban-Rural Resident Basic Medical Insurance.

Table S3. Medical behaviors of hypertensive patients aged 90 or older in Beijing, China.

| Medical behaviors | UEBMI | | URRBMI | | Total | |
| --- | --- | --- | --- | --- | --- | --- |
|  | Visit | % | Visit | % | Visit | % |
| Hospital level |  |  |  |  |  |  |
| Level 3 | 94307 | 28.20 | 8073 | 16.35 | 102380 | 26.68 |
| Level 2 | 35534 | 10.63 | 4111 | 8.33 | 39645 | 10.33 |
| Level 1 | 85807 | 25.66 | 18759 | 38.00 | 104566 | 27.25 |
| No level | 118727 | 35.51 | 18424 | 37.32 | 137151 | 35.74 |
| Hospital type |  |  |  |  |  |  |
| Comprehensive hospital | 183439 | 54.86 | 28888 | 58.52 | 212327 | 55.33 |
| Specialized hospital | 25025 | 7.48 | 1823 | 3.69 | 26848 | 7.00 |
| Traditional Chinese medicine hospital | 36532 | 10.93 | 2747 | 5.56 | 39279 | 10.24 |
| Community hospital | 89379 | 26.73 | 15909 | 32.23 | 105288 | 27.44 |
| Grassroots medical institution, yes | 172913 | 51.71 | 35828 | 72.57 | 208741 | 54.40 |
| Cross-district, yes | 132639 | 39.67 | 8271 | 16.75 | 140910 | 36.72 |

UEBMI, Urban Employee Basic Medical Insurance; URRBMI, Urban-Rural Resident Basic Medical Insurance.
